# Phylogeography above the species level for perennial species in a composite genus

**DOI:** 10.1093/aobpla/plv142

**Published:** 2015-12-07

**Authors:** Karin Tremetsberger, María Ángeles Ortiz, Anass Terrab, Francisco Balao, Ramón Casimiro-Soriguer, María Talavera, Salvador Talavera

**Affiliations:** 1 Departamento de Biología Vegetal y Ecología, Universidad de Sevilla, Apdo. 1095, 41080 Seville, Spain; 2 Present address: Institute of Botany, Department of Integrative Biology and Biodiversity Research, University of Natural Resources and Life Sciences, Gregor Mendel Straße 33, 1180 Vienna, Austria; 3 Present address: Departamento de Biología, CASEM, Universidad de Cádiz, Campus Río San Pedro, 11510 Puerto Real, Spain

**Keywords:** Amplified fragment length polymorphism, *Helminthotheca*, Iberian Peninsula, phylogeography, Strait of Gibraltar, western Mediterranean region, western North Africa

## Abstract

In phylogeography, DNA sequence and fingerprint data at the population level are used to infer evolutionary histories of species. Phylogeography above the species level is concerned with the genealogical aspects of divergent lineages. Here, we present a phylogeographic study to examine the evolutionary history of a western Mediterranean composite, focusing on the perennial species of *Helminthotheca* (Asteraceae, Cichorieae). We used molecular markers (amplified fragment length polymorphism (AFLP), internal transcribed spacer and plastid DNA sequences) to infer relationships among populations throughout the distributional range of the group. Interpretation is aided by biogeographic and molecular clock analyses. Four coherent entities are revealed by Bayesian mixture clustering of AFLP data, which correspond to taxa previously recognized at the rank of subspecies. The origin of the group was in western North Africa, from where it expanded across the Strait of Gibraltar to the Iberian Peninsula and across the Strait of Sicily to Sicily. Pleistocene lineage divergence is inferred within western North Africa as well as within the western Iberian region. The existence of the four entities as discrete evolutionary lineages suggests that they should be elevated to the rank of species, yielding *H. aculeata*, *H. comosa*, *H. maroccana* and *H. spinosa*, whereby the latter two necessitate new combinations.

## Abstract (precis)

Phylogeography above the species level is a powerful tool for investigating patterns and processes at the boundary between divergent and reticulate relationships. We examined the evolutionary history of perennial species in the western Mediterranean composite genus *Helminthotheca* using DNA sequence and fingerprint data. The origin of the group was in western North Africa, a region of intensive Pleistocene speciation. From here it expanded to the Iberian Peninsula and Sicily. The inferred evolutionary history is compatible with the concept of ecogeographic isolation, which refers to the fact that geographic ranges of diverging lineages are largely non-overlapping due to adaptive differentiation.

## Introduction

Intraspecific phylogeography as originally defined by Avise *et al*. (1987) is concerned with the geographic distribution of genealogical lineages at the level of conspecific populations. Originally based upon animal mitochondrial DNA sequence data, phylogeographic studies today make use of a variety of molecular techniques and are based upon DNA sequence and fingerprint data from the mitochondrial, plastid and nuclear genomes. The field of phylogeography today extends from the level of conspecific populations to the supraspecific level (Avise 2000) and targets organisms from the bacteria and eukaryote domains of life as well as viruses. Phylogeography of closely related species spans the boundary between reticulate and divergent relationships, where a partially braided collection of allelic pathways of interbreeding individuals bifurcates into two such collections (Avise 2000). Likewise, genealogical data at the population level of closely related species are especially helpful for explaining distributions resulting from historical and ecological factors.

A region that offered many opportunities for ecogeographic and effective geographic isolation (Sobel *et al*. 2010) due to its variegated geologic and climatic history since the Miocene is the western Mediterranean region. The formation of the Betic-Rif orogen led to the desiccation of the Mediterranean Sea between 6 and 5.3 million years ago (mya; Hsü *et al*. 1973), enabling the migration of animals and plants between the two continents (Bocquet *et al*. 1978). At the end of the Messinian, the Strait of Gibraltar opened and water from the Atlantic Ocean refilled the dry Mediterranean Basin (Lonergan and White 1997), thus again disconnecting north-western Africa from south-western Europe.

Major climatic events after the opening of the Strait of Gibraltar were the advent of the Mediterranean climate and Pleistocene climatic oscillations. In northern Africa, the appearance of a local or temporary dry season was already recognizable in the floristic composition of the Miocene and this climate trend was accentuated in the Pliocene (Quézel 1978). In the north-western Mediterranean area, a Mediterranean climatic rhythm with summer drought appeared in the Pliocene, at ∼3.2 mya (Suc 1984). In the Pleistocene, the water level of the Mediterranean Sea underwent great oscillations. During the last glacial maximum (0.02 mya), it was 120 m lower than today, so that the oceanic barrier between north-western Africa and south-western Europe was much reduced in extension, though not completely disrupted (Thiede 1978). The western Mediterranean region was an important refugium for temperate as well as Mediterranean plants and animals during the Pleistocene glacial periods (Médail and Diadema 2009). It is supposed that the changing environmental conditions and appearance/disappearance of biogeographic barriers have massively triggered plant and animal diversification in the Pliocene and Pleistocene (Fiz-Palacios and Valcárcel 2013).

The genus *Helminthotheca* (Asteraceae, Cichorieae, Hypochaeridinae) has a primarily western Mediterranean distribution. It has been separated from *Picris* at the generic level on the basis of the conspicuously enlarged outer involucral bracts and comprises the annual, heterocarpic species *H. echioides* and *H. balansae* and the perennial, homocarpic species *H. aculeata*, *H. comosa* and *H. glomerata* (*Picris comosa* auct. alg.; Lack 1974, 1975). *Helminthotheca aculeata* has another subspecies, *H. aculeata* subsp. *maroccana*, in addition to the nominal subspecies. Similarly, *H. comosa* also has another subspecies, *H. comosa* subsp. *lusitanica*, in addition to the nominal subspecies.

The most recent common ancestor (MRCA) of extant taxa of *Helminthotheca* can be traced back to the Pliocene or Pleistocene (Tremetsberger *et al*. 2013). It is thus likely that populations have been affected by climatic changes occurring in the Mediterranean region in this epoch, possibly leading to range shifts, disruptions of previously continuous ranges and secondary contacts. This study aims at shedding light on the evolutionary history of the perennial species of *Helminthotheca*. Using molecular makers from the nuclear and plastid genomes, we addressed the following questions: (i) Does genetic grouping correlate with the present taxonomic treatment? (ii) Where did perennial species of *Helminthotheca* originate? (iii) Along which routes did they spread to occupy their present areas? The inferred evolutionary history is discussed with respect to the evolutionary forces that might have been relevant for speciation.

## Methods

### Plant material

**Figure 1.**
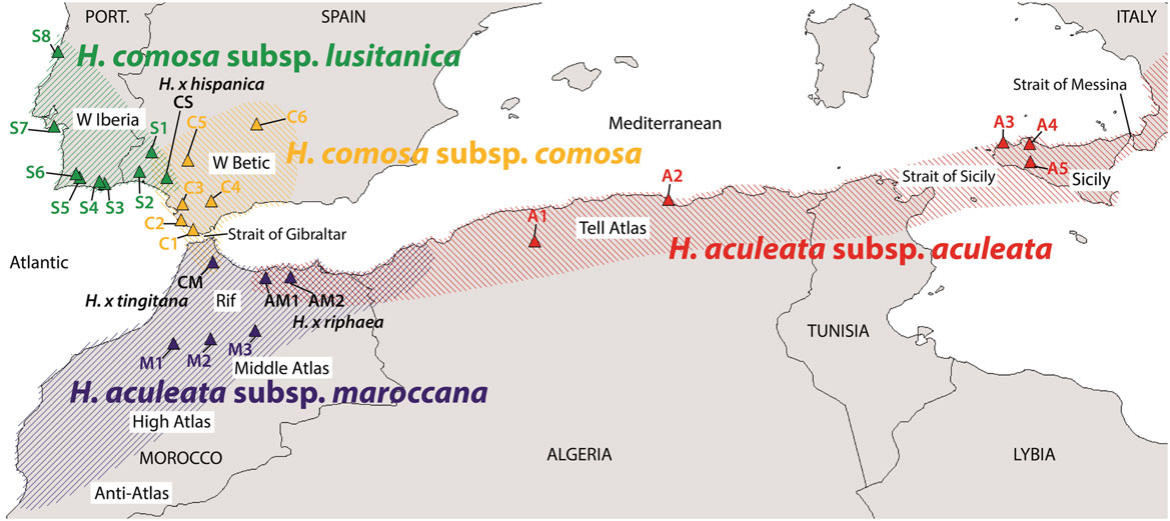
Distribution ranges of *H. aculeata* subsp*. aculeata*, *H. aculeata* subsp. *maroccana* (*H. maroccana*, comb. nov.), *H. comosa* subsp. *comosa* and *H. comosa* subsp. *lusitanica* (*H. spinosa*, comb. nov.) and populations analysed in this study [colour of triangles indicates belonging of populations to one of four clusters inferred by Bayesian mixture clustering of AFLP data (see Fig. 2)].

We sampled populations belonging to subspecies of *Helminthotheca aculeata* and *H. comosa* in Algeria, Italy (Sicily), Morocco, Portugal and Spain (Table 1, Fig. 1). *Helminthotheca echioides* was included as outgroup. Vouchers of all populations used are deposited in the herbaria at Seville (SEV) and/or Vienna (WU). The Algerian species *H. balansae* and *H. glomerata* (Quézel and Santa 1963) could not be sampled for this study. In the field, leaves from individual plants were conserved in silica gel. Distribution ranges of the taxa under study were compiled from survey of material in the herbaria at Barcelona (BC), Geneva (G), Madrid (MA), Montpellier (MPU), Paris (P), Salamanca (SALA) and Seville (SEV). Locations mentioned in Pignatti (1982) and Sauvage (1961) were also used. DIVA-GIS ver. 7.1.7.2 (Hijmans *et al*. 2001) was used to display distribution ranges and sample locations.

**Table 1.** Population samples of *Helminthotheca* used in this study with EMBL accession numbers of DNA sequences.

Taxon with population code	Locality, collector(s) and number	EMBL accession numbers		
		ITS	*ndhF-rpl32*	*rpl32-trnL*
*Helminthotheca aculeata* (Vahl) Lack subsp. *aculeata*				
Pop. A1	Algeria, Tissemsilt Province (Tell Atlas): Theniet-el-Had National Park (35.85°N, 1.98°E), *Véla s/n* (9 June 2006)	LN830805	LN830890	LN830846
Pop. A2	Algeria, Béjaïa Province (Tell Atlas): Gouraya National Park, *Véla s/n* (2 July 2008)	LN830806	LN830891	LN830847
Pop. A3	Italy, Sicilian Region: Monte Cofano NW of Custonaci (38.10°N, 12.67°E), *Ortiz & Tremetsberger 24/08*	LN830807 (two identical individuals)	LN830892 (two identical individuals)	LN830848, LN830849
Pop. A4	Italy, Sicilian Region: Castellaccio W of Monreale (38.08°N, 13.27°E), *Ortiz & Tremetsberger 27/08*	LN830808, LN830809	LN830893, LN830894	LN830850, LN830851
Pop. A5	Italy, Sicilian Region: Chiusa Sclafani (37.66°N, 13.28°E), *Ortiz & Tremetsberger 30/08*	LN830810, LN830811	LN830895 (three identical individuals)	LN830852, LN830853 (two identical individuals)
*Helminthotheca aculeata* subsp. *maroccana* (Sauvage) Greuter [*H. maroccana* (Sauvage) Talavera & Tremetsberger, comb. nov.]				
Pop. M1	Morocco, Rabat-Salé-Zemmour-Zaer Region (Middle Atlas): Montes de Zaïan between Tiddas and Oulmes (33.52°N, 6.26°W), *Talavera et al. 171/06*	LN830799, LN830800	LN830886, LN830887	LN830842, LN830843
Pop. M2	Morocco, Meknès-Tafilalet Region (Middle Atlas): S of Bou Fekrane on road to Jemaa de Mrirt (33.62°N, 5.43°W), *Talavera et al. 796/05*	LN830797, LN830798	LN830885 (two identical individuals)	LN830840, LN830841
Pop. M3	Morocco, Fès-Boulemane Region (Middle Atlas): Ribate El Kheir (33.82°N, 4.41°W), *Talavera et al. 37/08*	LN830795, LN830796	LN830882, LN830883, LN830884	LN830837, LN830838, LN830839
*H. comosa* (Boiss.) Holub subsp. *comosa*				
Pop. C1	Spain, Cádiz Province (W Betic): Zahara de los Atunes (36.11°N, 5.82°W), *König s/n* (22 May 2008)	LN830782, LN830783	LN830875 (three identical individuals)	LN830831 (three identical individuals)
Pop. C2	Spain, Cádiz Province (W Betic): Conil de la Frontera (36.33°N, 6.09°W), *Talavera et al. 11/08*	LN830789, LN830790, LN830791, LN830792	LN830879 (three identical individuals)	LN830835 (three identical individuals)
Pop. C3	Spain, Cádiz Province (W Betic): Jerez de la Frontera (36.69°N, 6.06°W), *Sánchez s/n* (30 July 2007)	LN830786, LN830787, LN830788	LN830877 (two identical individuals), LN830878	LN830833 (two identical individuals), LN830834
Pop. C4	Spain, Cádiz Province (W Betic): Grazalema (36.76°N, 5.40°W), *Talavera & Talavera 112/08*	LN830784, LN830785	LN830876 (three identical individuals)	LN830832 (three identical individuals)
Pop. C5	Spain, Seville Province (W Iberia): Castilblanco de los Arroyos (37.70°N, 5.94°W), *Talavera et al. 7/06*	LN830778, LN830779	LN830873 (three identical individuals)	LN830829 (three identical individuals)
Pop. C6	Spain, Ciudad Real Province (W Iberia):: Puertollano (38.52°N, 4.37°W), *Talavera et al. 205/06*	LN830780, LN830781	LN830874 (three identical individuals)	LN830830 (three identical individuals)
*H. comosa* subsp. *lusitanica* (Welw. ex Schltdl.) P. Silva & Escud. [*H. spinosa* (DC.) Talavera & Tremetsberger, comb. nov.]				
Pop. S1	Spain, Huelva Province (W Iberia): Almonaster la Real (37.88°N, 6.77°W), *Talavera et al. 324/06*	LN830774, LN830775	LN830870 (two identical individuals), LN830871	LN830825 (three identical individuals)
Pop. S2	Spain, Huelva Province (W Iberia): between Gibraleón and San Bartolomé de la Torre (37.43°N, 7.05°W), *Tremetsberger & Ortiz 1/06*	LN830776, LN830777	LN830872 (three identical individuals)	LN830826, LN830827, LN830828
Pop. S3	Portugal, Faro District (W Iberia): between Sao Bras de Alportel and Santa Catarina (37.15°N, 7.83°W), *Talavera et al. 214/06*	LN830760, LN830761	LN830858 (three identical individuals)	LN830816 (three identical individuals)
Pop. S4	Portugal, Faro District (W Iberia): between Querenca and Barranco do Velho (37.21°N, 7.96°W), *Talavera et al. 219/06*	LN830770, LN830771	LN830866, LN830867 (two identical individuals)	LN830823 (three identical individuals)
Pop. S5	Portugal, Faro District (W Iberia): between Silves and Sao Marcos da Serra (37.30°N, 8.40°W), *Talavera et al. 224/06*	LN830762, LN830763	LN830859 (two identical individuals), LN830860	LN830817 (two identical individuals), LN830818
Pop. S6	Portugal, Beja District (W Iberia): Serra de Monchique between Monchique and Odemira (37.39°N, 8.49°W), *Talavera et al. 244/06*	LN830764, LN830765	LN830861 (two identical individuals), LN830862	LN830819 (three identical individuals)
Pop. S7	Portugal, Setubal District (W Iberia): Serra da Arrábida between Setubal and Portinho da Arrábida (38.47°N, 9.00°W), *Talavera et al. 262/06*	LN830766, LN830767	LN830863 (three identical individuals)	LN830820 (three identical individuals)
Pop. S8	Portugal, Coimbra District (W Iberia): Figueira da Foz, Cabo Mondego (40.18°N, 8.90°W), *Talavera et al. 263/06*	LN830768, LN830769	LN830864 (two identical individuals), LN830865	LN830821 (two identical individuals), LN830822
*Helminthotheca echioides* (L.) Holub				
Pop. E1	Portugal, Coimbra District: Figueira da Foz, Cabo Mondego (40.18°N, 8.90°W), *Talavera et al. 266/06*	LN830755	LN830854	LN830812
Pop. E2	Spain, Huelva Province: between Gibraleón and Cartaya (37.33°N, 7.03°W), *Tremetsberger & Ortiz 2/06*	LN830756	LN830855	LN830813
Pop. E3	Spain, Cádiz Province: El Palmar (36.32°N, 6.08°W), *Talavera & Ortiz s/n* (26 April 2006)	LN830757	LN830856	LN830814
Pop. E4	Morocco, Tangier-Tetouan Region: S of Ksar es-Seghir (35.77°N, 5.53°W), *Talavera et al. 23/07*	LN830758 (four identical individuals)	–	–
Pop. E5	Algeria, Annaba Province: El Kerma on road Annaba-Guelma (36.70°N, 7.62°E), *Véla s/n* (5 June 2006)	LN830759	LN830857	LN830815
*Helminthotheca* × *hispanica* Tremetsberger & Talavera, hybr. nov.				
Pop. CS	Spain, Huelva Province (W Iberia): Hinojos (37.29°N, 6.42°W), *Talavera 228/07*	LN830772, LN830773	LN830868, LN830869 (two identical individuals)	LN830824 (three identical individuals)
*Helminthotheca* × *riphaea* Tremetsberger & Talavera, hybr. nov.				
Pop. AM1	Morocco, Taza-Al Hoceima-Taounate Region (Rif): between Targuist and Al Hoceima (35.02°N, 4.17°W), *Rico et al. MS-1099*	LN830801, LN830802	LN830888 (three identical individuals)	LN830844 (three identical individuals)
Pop. AM2	Morocco, Taza-Al Hoceima-Taounate Region (Rif): between Tafersit and Temsaman (35.04°N, 3.59°W), *Rico et al. MS-1145*	LN830803, LN830804	LN830889 (three identical individuals)	LN830845 (three identical individuals)
*Helminthotheca* × *tingitana* Tremetsberger & Talavera, hybr. nov.				
Pop. CM	Morocco, Tangier-Tetouan Region (Rif): between Chefchaouen and Tetouan (35.37°N, 5.38°W), *Rico et al. MS-999*	LN830793, LN830794	LN830880 (two identical individuals), LN830881	LN830836 (three identical individuals)

### Amplified fragment length polymorphism (AFLP)

The protocol for extraction of genomic DNA from silica gel-dried leaves, restriction-ligation (*Eco*RI and *Mse*I), preselective and selective amplification, and electrophoresis on a capillary sequencer (3130*xl* Genetic Analyzer, Applied Biosystems) exactly followed Tremetsberger *et al*. (2009). Three selective primer combinations [*Eco*RI(Fam)-ACA/*Mse*I-CAT, *Eco*RI(Vic)-AGG/*Mse*I-CTC and *Eco*RI(Ned)-AAC/*Mse*I-CTT] were selected following a primer trial testing 12 selective primer combinations, based on the criteria of interpretability (presence/absence), spread of bands in the region 50–500 bp, and number of bands (∼70–90 per primer combination). Raw data were aligned with the GeneScan 500 ROX size standard using ABI PRISM GeneScan ver. 3.71 (©Applied Biosystems). The presence/absence of bands in all individuals and replicates was scored with Genographer ver. 1.6.0 (Benham 2001) after normalizing on total signal in one file. We scored 96 individuals from 25 populations of the perennial species of *Helminthotheca*. Twenty-two individuals were replicated from restriction-ligation and used to calculate the error rate as the ratio between observed number of phenotypic differences and total number of phenotypic comparisons between replicates (Bonin *et al*. 2004). Three individuals of *H. echioides* were used as outgroup. Only loci observed in the ingroup were scored in the outgroup.

We employed split decomposition (Bandelt and Dress 1992) for constructing a phylogenetic network, which is preferred over a strictly bifurcating tree at the population level, where gene flow is to be expected. FAMD ver. 1.108 (Schlüter and Harris 2006) was used to calculate a matrix of Nei and Li (1979) distances with the length of the restriction enzyme's recognition sequence set to six between all pairs of individuals. This matrix was imported into SplitsTree ver. 4.8 (Huson and Bryant 2006) to construct a NeighborNet network. Splits, whose weight did not exceed a threshold of 0.001, were removed.

Bayesian admixture clustering (Corander *et al*. 2003; Corander and Marttinen 2006) was used to estimate the number of genetically coherent clusters in the perennial species of *Helminthotheca* with BAPS ver. 5.2 (Corander *et al*. 2004, 2008). The maximum number of populations for mixture clustering was set to 20 (5×), 25 (5×) and 30 (5×). For admixture clustering based on results of mixture clustering, the minimum size of a population was set to 1, the number of iterations to 200 and the number of reference individuals to 200 with 20 iterations each.

In order to determine which taxon might be ancestral, we generated a phylogenetic tree, which also included three individuals of the outgroup, *H. echioides*, by neighbour-joining analysis based on Nei-Li distances in PAUP* ver. 4.0b10 (Swofford 2003). The support of nodes was estimated by 500 bootstrap replicates. Clusters containing only individuals of the same population or taxon were collapsed using FigTree ver. 1.3.1 (Rambaut 2006–2009).

In order to distinguish between incongruent species trees, we adopted the approach of Bryant *et al*. (2012) to infer species trees from AFLP data in the framework of the multispecies coalescent, implemented in SNAPP (‘SNP and AFLP Phylogenies’), a Bayesian MCMC sampler, which interfaces with the BEAST package (Drummond and Rambaut 2007). Nine individuals per taxon were included in the analysis: *H. aculeata* subsp. *aculeata*, A1–A5; *H. aculeata* subsp. *maroccana*, M1–M3; *H. comosa* subsp. *comosa*, C2–C6; and *H. comosa* subsp. *lusitanica*, S1–S3, S5 and S6. Individuals of putative hybrid origin (see below) were not included. We ran two separate Markov chain Monte Carlo (MCMC) chains, each with 7 million states, without correction for dominance, in BEAST ver. 2.1.3 (Bouckaert *et al*. 2002–2014). Trees and associated parameter values were logged every 1000 states. Results of the MCMC runs were analysed with Tracer ver. 1.5.0 (Rambaut and Drummond 2003–2009), discarding the first 10 % as burn-in. Trees created in the two MCMC runs were combined with LogCombiner ver. 2.1.3, again discarding the first 10 % of each run as burn-in. A species tree was drawn from the combined trees with DensiTree ver. 2.1.11 (Bouckaert 2010).

Within-population genetic diversity was calculated as ‘average gene diversity over loci’ in Arlequin ver. 3.5.1.2 (Excoffier *et al*. 2005; only for populations with a minimum of three individuals). As indicators of divergence due to long-lasting isolation of populations, we estimated the number of private AFLP bands in a population (i.e. those bands occurring exclusively in the respective population) and the rarity index. Only populations with a minimum of two individuals were used. Furthermore, some populations with more than two individuals were also omitted, so that a similar number of populations was included for each taxon. The number of private bands was counted with FAMD ver. 1.25. The rarity index equivalent to frequency-down-weighted marker values (DW) of Schönswetter and Tribsch (2005) was calculated as ‘rarity 1’ using the R-script AFLPdat (Ehrich 2006) in R ver. 2.11.1 (©The R Foundation for Statistical Computing). For each individual, each AFLP band was divided by the total number of occurrences of this band in the dataset. These relative values were then summed to the rarity index for this particular individual. Population values were calculated as averages of individual values.

### DNA sequences

The internal transcribed spacer (ITS) region of nuclear ribosomal DNA was amplified by using primers ITS4 and ITS5 of White *et al*. (1990). The PCR mix for amplification (total volume 20 µL) contained: 18 µL 1.1× Reddy Mix PCR Master Mix (2.5 mM MgCl_2_; ABgene), 0.4 µL forward and reverse primer [20 µM] each, 0.8 µL dimethyl sulfoxide and 0.4 µL DNA extract. Amplification was performed with a Mastercycler (Eppendorf) under the following reaction conditions: 95 °C/4.5 min, 45 °C/1 min, 72 °C/1 min (1 cycle); 95 °C/1 min, 48 °C/1 min, 72 °C/1 min (36 cycles); and 72 °C/7 min, 4 °C/thereafter. Amplification products were purified by enzyme treatment with 0.5 µL Exonuclease I and 1 µL Calf Intestine Alkaline Phosphatase (Fermentas; incubation at 37 °C for 45 min followed by enzyme inactivation at 85 °C for 15 min) and cycle sequenced on a GeneAmp PCR System 9700 (Applied Biosystems) by following the ABI PRISM BigDye Terminator Cycle Sequencing Ready Reaction Kit Protocol (Applied Biosystems). As a modification, the total volume of the cycle sequencing reaction was 10 µL (1 µL Terminator Ready Reaction Mix, 1.5 µL 5× sequencing buffer [10 mM], 1 µL forward or reverse primer [5 µM], and 6.5 µL purified amplification product). Unincorporated dye terminators were removed by centrifugation through MultiScreen-HV filter plates (Millipore) filled with Sephadex G-50 Fine (GE Healthcare) prior to electrophoresis on a 3130xl Genetic Analyzer (Applied Biosystems).

We tested several plastid intergenic spacer regions, namely *atpI-atpH*, *ndhF-rpl32*, *petL-psbE*, *psbD-trnT*, *psbJ-petA*, *rpl32-trnL*, *3′rps16-5′trnK*, *trnQ-5′rps16*, and *3′trnV-ndhC* (Shaw *et al*. 2007) and *psbA-trnH* (Sang *et al*. 1997), for their phylogenetic information content in *Helminthotheca*. The two most variable regions, *ndhF-rpl32* and *rpl32-trnL*, were sequenced in a larger number of individuals. Addition of the remaining regions did not substantially improve phylogenetic resolution. The PCR mix for amplification (total volume 18 µL) contained: 14.4 µL 1.1× Reddy Mix PCR Master Mix (2.5 mM MgCl_2_; ABgene), 0.9 µL forward and reverse primer [10 µM] each, 0.9 µL 0.4 % bovine serum albumin (BSA) and 0.9 µL DNA extract. Amplification was performed under the following reaction conditions: 80 °C/5 min; 95 °C/30 s, 50 °C/30 s, 65 °C/4 min (36 cycles); and 65 °C/5 min, 4 °C/thereafter. Amplification products were purified by enzyme treatment with 0.5 µL Exonuclease I and 1 µL FastAP Thermosensitive Alkaline Phosphatase (Fermentas; same conditions as for ITS) and cycle sequenced by following the ABI PRISM BigDye Terminator Cycle Sequencing Ready Reaction Kit Protocol (Applied Biosystems) in a 10 µL total reaction volume (0.6 µL Terminator Ready Reaction Mix, 1.7 µL 5 × sequencing buffer [10 mM], 1 µL forward or reverse primer [3.2 µM] and 6.7 µL purified amplification product). Products of the cycle sequencing reaction were purified with Sephadex G-50 Fine and run on a capillary sequencer (as for ITS).

Forward and reverse sequences were assembled with SeqMan II ver. 5.05 (DNASTAR, Inc., Madison, WI, USA). The sequences were aligned manually in BioEdit ver. 7.0.5.3 (Hall 1999). SeqScape ver. 2.5.0 (Applied Biosystems) was used to check all mutations in the alignments as well as multiple states (additivities) found in many ITS sequences. Because the peaks of the two bases contained in the multiple states had similar heights, they were interpreted as real polymorphisms due to incomplete concerted evolution and not as sequencing uncertainties. BioEdit was also used to calculate G + C contents of ITS sequences. We also reconstructed the secondary structure of ITS2 of the perennial species of *Helminthotheca*, using the RNA folding form of mfold ver. 3.5 (Zuker 2003), based on the common structure described for Asteraceae by Goertzen *et al*. (2003). Sequences were deposited in the EMBL Nucleotide Sequence Database (Stoesser *et al*. 2003; Table 1).

For phylogenetic analysis of ITS sequences, we adopted the maximum likelihood (ML) approach using the RAxML software on the Vital IT unit of the Swiss Institute of Bioinformatics Web server (http://embnet.vital-it.ch/raxml-bb/index.php, 24 July 2015; Stamatakis *et al*. 2008), with individual general time reversible (GTR) models of nucleotide substitution with gamma-distributed site-to-site rate variation and a proportion of invariable sites being estimated for the three partitions ITS1, 5.8S rDNA and ITS2. Support values were obtained by bootstrapping (100 replicates).

For analysis of concatenated *ndhF-rpl32* and *rpl32-trnL* sequences, the maximum parsimony (MP) approach was adopted due to its ease of incorporating information contained in indels, which were coded using the simple indel coding method implemented in SeqState ver. 1.3.2 (Müller 2005). A heuristic search under the MP criterion was carried out with 1000 random-addition-sequence replicates and TBR swapping with no more than 10 trees of score ≥10 retained in each replicate with PAUP* ver. 4.0b10 (Swofford 2003). Bootstrap support was estimated with 500 bootstrap replicates (Felsenstein 1985; heuristic search with 10 random-addition-sequence replicates and TBR swapping with no more than 10 trees of score ≥10 retained in each replicate).

A species tree was constructed from ITS and plastid DNA sequences (without indel coding) in BEAST ver. 2.1.3 using the *BEAST template (Heled and Drummond 2010), with a Jukes-Cantor (JC69) site model, a relaxed log normal clock model, a birth-death tree model with constant population size and 10 million states of the MCMC chain. Individuals of putative hybrid origin (see below) were not included. Populations were assigned to the following taxa: *H. aculeata* subsp. *aculeata*, A1–A5; *H. aculeata* subsp. *maroccana*, M1–M3 (the plastid DNA sequences of two individuals that have the ‘comosa/lusitanica’-plastid type [populations M1 and M3; see below] have not been included); *H. comosa* subsp. *comosa*, C1–C6; *H. comosa* subsp. *lusitanica*, S1–S8; and *H. echioides*, E1–E5. Use of other models (e.g. the GTR site model) did not affect the result. Trees were displayed in DensiTree ver. 2.1.11 (10 % burn-in).

### Biogeographic analysis

Ancestral areas were reconstructed by ML-based biogeographic analysis implemented in the Lagrange version released on 26 May 2013 (Ree and Smith 2008). The species tree generated from DNA sequences was used as input. Taxa were assigned to the geographic areas western North Africa (Rif and Middle Atlas; all taxa except *H. comosa* subsp. *lusitanica*), central North Africa (Tell Atlas; both subspecies of *H. aculeata*), Sicily (*H. aculeata* subsp. *aculeata*), the W Betic region (García-Barros *et al*. 2002; *H. comosa* subsp. *comosa*), and the W Iberian region (García-Barros *et al*. 2002; both subspecies of *H. comosa*). *Helminthotheca echioides* occurs in all areas. The maximum number of areas in ancestral ranges was set to 3. The area adjacency matrix is detailed in Fig. 5A. The dispersal rate was set to 1.0 between adjacent areas and 0.1 between non-adjacent areas.

### Molecular clock

We adopted the approach of Tremetsberger *et al*. (2013) for dating the age of the perennial species of *Helminthotheca*. The ITS1 and ITS2 alignments of Cichorieae (Tremetsberger *et al*. 2013; available from TreeBASE) and of *Helminthotheca* were merged. To simplify the calculation, some sequences were removed; totals of retained sequences were 25 for *Helminthotheca* and 46 for Cichorieae and outgroup taxa. BEAST ver. 1.6.2 was used as outlined in Tremetsberger *et al*. (2013) with stem group node calibration. Results of four independent MCMC runs were analysed with Tracer ver. 1.5.0 and combined with LogCombiner ver. 1.6.2 (10 % burn-in). The maximum clade credibility tree was obtained using TreeAnnotator ver. 1.6.2 and graphed with FigTree ver. 1.3.1.

## Results

### AFLP

**Figure 2.**
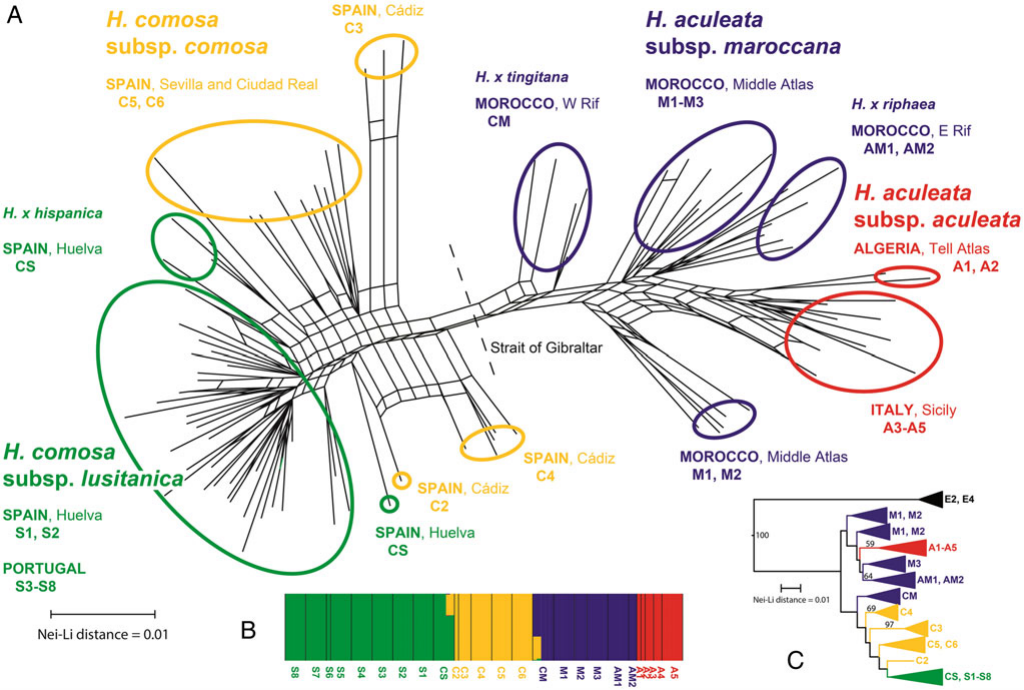
Results of AFLP analysis showing division of population samples of perennial species of *Helminthotheca* into four clusters. (A) Phylogenetic network (colour coding according to Bayesian mixture clustering). (B) Bayesian admixture clustering with evidence for admixture in populations CM and CS. (C) Neighbour-joining tree including the outgroup *H. echioides* (colour coding according to Bayesian mixture clustering). Numbers above branches indicate bootstrap support values >50 %.

In total, 231 AFLP bands in the range of 90–430 bp were scored for the 96 individuals of the perennial species of *Helminthotheca*. Of these, 207 (i.e. 90 %) are polymorphic. All individuals have unique AFLP phenotypes. The overall error rate was 4 % (ranging from 0 to 41 % for individual loci). Removal of seven loci with individual error rates >25 % did not significantly alter the phylogenetic network (Fig. 2A). We thus decided to rely on the original matrix for all further calculations.

In the Bayesian mixture clustering analysis, the first nine best visited partitions revealed four clusters, whereas the 10th best visited partition revealed five clusters. The values for the log of the marginal likelihood of the nine optimal partitions range from −5916 to −5960 (the 10th best visited partition has a value of −5971). The posterior probability for *K* = 4 is 1. The four clusters revealed (Figs 1 and 2) are (i) populations A1–A5 of *H. aculeata* subsp. *aculeata* (Algeria, Sicily), (ii) populations M1–M3 of *H. aculeata* subsp. *maroccana* together with populations AM1 and AM2, which morphologically resemble *H. aculeata* subsp. *aculeata*, and population CM, which morphologically resembles *H. comosa* subsp. *comosa* (all Morocco), (iii) populations C2–C6 of *H. comosa* subsp. *comosa* (Spain), and (iv) populations S1–S8 of *H. comosa* subsp. *lusitanica* (Portugal, Spain) together with population CS (Spain). Some evidence for admixture was also found, namely in some individuals of populations CM and CS, which show admixture from the *H. comosa*-cluster (Fig. 2B).

Network analysis (Fig. 2A) shows relationships among individuals of the four clusters. The major split is across the Strait of Gibraltar separating the *H. comosa* subsp. *comosa*/*H. comosa* subsp. *lusitanica*-cluster from the *H. aculeata* subsp. *maroccana*/*H. aculeata* subsp. *aculeata*-cluster. Within the Afro-Sicilian group, the *H. aculeata* subsp. *maroccana*-populations M1 and M2 from the Middle Atlas branch off basally, whereas the *H. aculeata* subsp. *aculeata*-populations (A1–A5) are derived (Fig. 2C). Within the Iberian group, the *H. comosa* subsp. *comosa*-populations (C2–C6) branch off basally, whereas the *H. comosa* subsp. *lusitanica*-populations (S1–S8) are derived.

**Figure 3.**
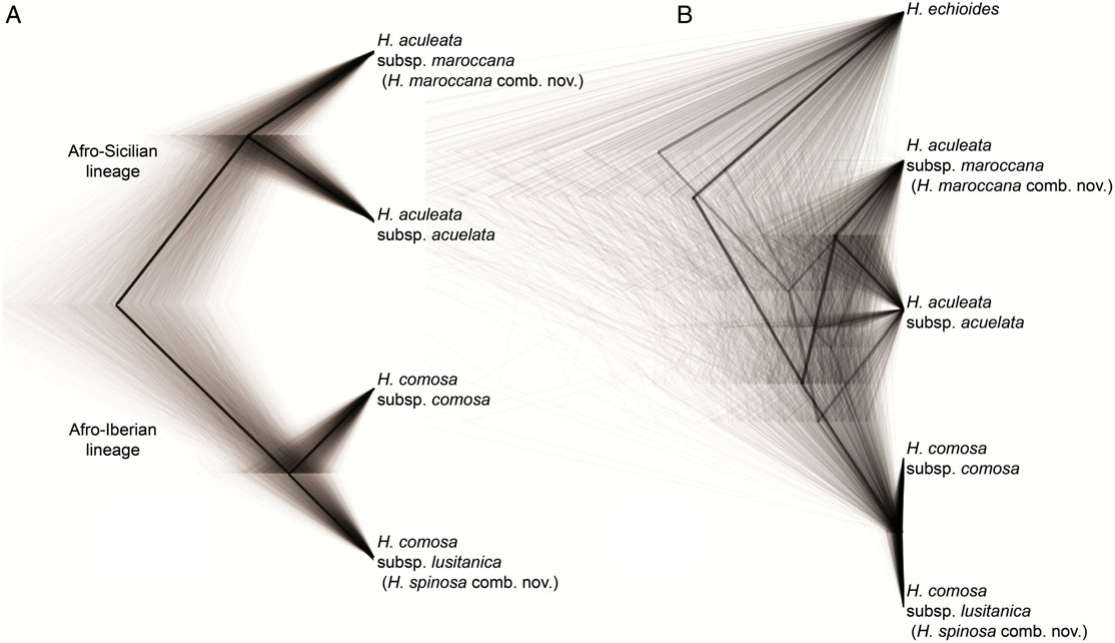
DensiTree of the perennial clade of *Helminthotheca* and the outgroup *H. echioides*, showing the complete tree set (thin lines) and consensus trees (thick lines; 10 % burn-in) from (A) SNAPP analysis of AFLP data (single consensus tree) and (B) *BEAST analysis of ITS and plastid DNA sequences. The posterior probability of the *H. aculeata* subsp. *maroccana*/*H. aculeata* subsp. *aculeata*-clade is 58 %. With coded indels of plastid DNA sequences included, it increases to 92 % (not shown).

Evaluation of sampled trees from the coalescent approach to species tree reconstruction revealed an effective sample size (ESS) of >10 000 for the two combined runs. The traces did not show signs of non-convergence. All clades have posterior probabilities of 100 %, yielding a single consensus tree (Fig. 3A). In a tree lacking short branches, results of analyses with and without correction for dominance do not differ (Bryant *et al*. 2012), so that we relied only on the analysis without correction for dominance. The sequence of divergence suggested by the obtained species tree is: first, divergence of an Afro-Sicilian and an Afro-Iberian lineage; second, divergence of *H. aculeata* subsp. *maroccana* and *H. aculeata* subsp. *aculeata* in the Afro-Sicilian lineage; and third, divergence of *H. comosa* subsp. *comosa* and *H. comosa* subsp. *lusitanica* in the Afro-Iberian lineage.

Accumulation of private and rare AFLP bands in populations due to mutations indicates long-term *in situ* persistence of populations. This is in contrast to newly established populations, which should have fewer such bands. The mean number of private bands and the rarity index are slightly higher in populations of the Afro-Sicilian lineage than in populations of the Afro-Iberian lineage (Table 2). This trend is accentuated, when the estimates are calculated for taxa rather than for populations. *Helminthotheca aculeata* subsp. *aculeata* (populations A1–A5 [*N*_individuals_ = 11]) has 20 private bands and a rarity index of 4.1. Similarly, *H. aculeata* subsp. *maroccana* (populations M1–M3 [*N*_individuals_ = 13]) has 19 private bands and a rarity index of 4.1. *Helminthotheca comosa* subsp. *comosa* (populations C3–C6 [*N*_individuals_ = 18]) has 16 private bands and a rarity index of 2.6 and *H. comosa* subsp. *lusitanica* (populations S2, S5, S7, and S8 [*N*_individuals_ = 20]) has 12 private bands and rarity index of 2.2. In parallel to estimates of divergence, within-population genetic diversity is also slightly higher in populations of the Afro-Sicilian lineage than in populations of the Afro-Iberian lineage (Table 2).

**Table 2.** Estimates of divergence and diversity derived from AFLP data in populations of *Helminthotheca. N*, sample size; ND, not determined.

Species and region	*N*	Estimates of divergence		Within-population genetic diversity
Population		*N*_private bands_	Rarity index	
*H. aculeata* (Vahl) Lack subsp. *aculeata*, Algeria and Sicily				
A3	2	2	3.5	ND
A4	2	2	3.0	ND
A5	5	6	3.3	0.099
Mean ± SD		3.3 ± 2.3	3.3 ± 0.3	0.099
*H.* × *riphaea* Tremetsberger & Talavera, hybr. nov., Morocco				
AM1	5	3	2.9	0.098
AM2	2	9	7.4	ND
Mean ± SD		6.0 ± 4.2	5.2 ± 3.2	0.098
*H. aculeata* subsp. *maroccana* (Sauvage) Greuter [*H. maroccana* (Sauvage) Talavera & Tremetsberger, comb. nov.], Morocco				
M1	5	1	2.3	0.113
M2	3	4	3.3	0.115
M3	5	4	4.3	0.121
Mean ± SD		3.0 ± 1.7	3.3 ± 1.0	0.116 ± 0.004
*H.* × *tingitana* Tremetsberger & Talavera, hybr. nov., Morocco				
CM	5	1	2.5	0.109
*H. comosa* (Boiss.) Holub subsp. *comosa*, Spain				
C3	3	7	4.5	0.069
C4	5	3	2.3	0.068
C5	5	0	1.4	0.104
C6	5	3	2.1	0.097
Mean ± SD		3.3 ± 2.9	2.6 ± 1.3	0.084 ± 0.019
*H.* × *hispanica* Tremetsberger & Talavera, hybr. nov., Spain				
CS	5	ND	ND	0.100
*H. comosa* subsp. *lusitanica* (Welw. ex Schltdl.) P. Silva & Escud. [*H. spinosa* (DC.) Talavera & Tremetsberger, comb. nov.], Spain and Portugal				
S1	5	ND	ND	0.103
S2	5	1	1.9	0.096
S3	5	ND	ND	0.080
S4	5	ND	ND	0.086
S5	5	5	2.7	0.097
S7	5	0	1.7	0.055
S8	5	3	2.2	0.083
Mean ± SD		2.3 ± 2.2	2.1 ± 0.5	0.087 ± 0.015

### DNA sequences

The G+C content of ITS1 and ITS2 sequences is very similar among accessions of the perennial species of *Helminthotheca* (mean = 53.3 %; SD = 0.2 %) and *H. echioides* (mean = 54.0 %; SD = 0.3 %), suggesting the absence of pseudogenes. The ITS2 secondary structure is common to all individuals of *Helminthotheca* analysed **[see** **Supporting Information—Fig. S1****]**. It is also well in accordance with the ITS2 secondary structure of Asteraceae presented by Goertzen *et al*. (2003). No compensatory base changes (Coleman 2007) exist among individuals of *Helminthotheca*, neither when comparisons are made among perennial species nor when these are compared with *H. echioides*.

**Figure 4.**
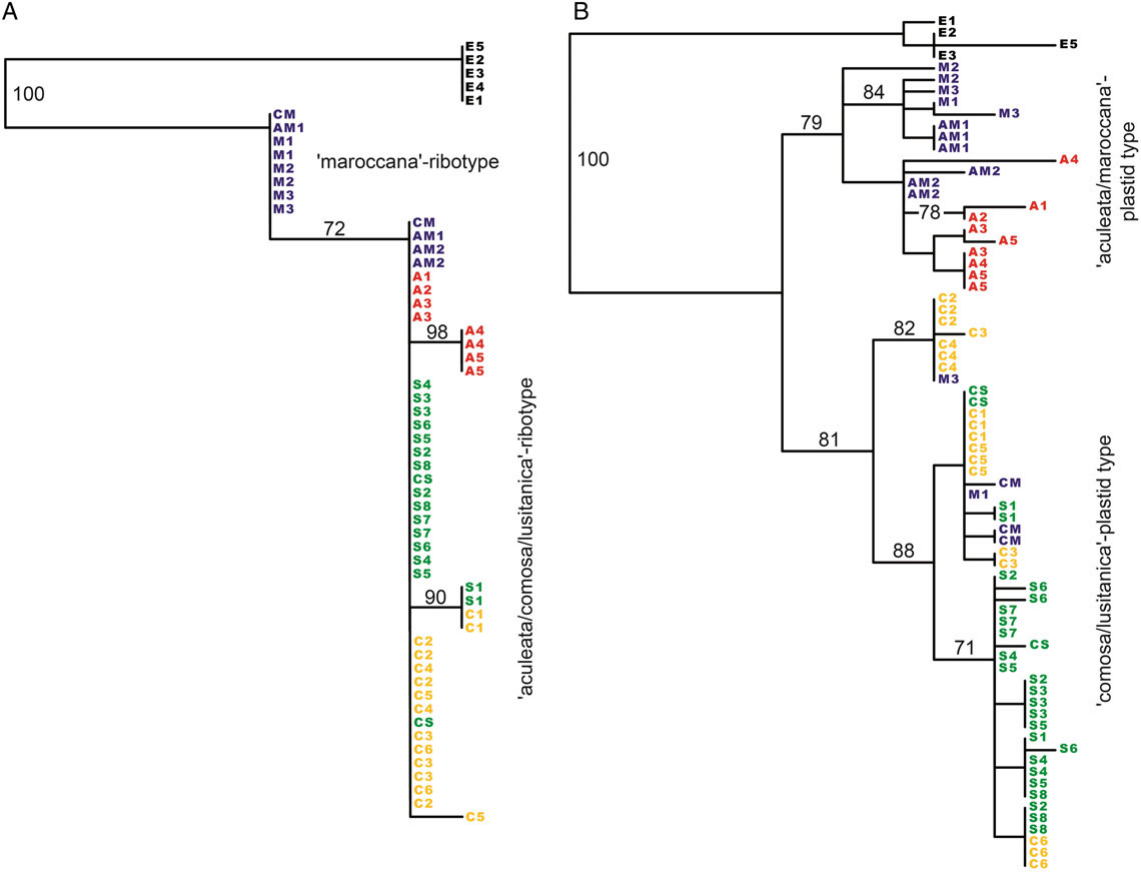
Phylogenetic trees of perennial species of *Helminthotheca*, with *H. echioides* designated as outgroup. (A) Best-scoring ML tree of ITS1, 5.8S rDNA and ITS2 sequences. (B) Bootstrap 50 % majority rule consensus tree (plus other groups compatible with it) based on MP analysis of concatenated *ndhF-rpl32* and *rpl32-trnL* sequences. Numbers above branches indicate bootstrap support values >70 %. Colour coding indicates belonging of individuals to one of four clusters inferred by Bayesian mixture clustering of AFLP data (see Fig. 2).

The ITS tree (Fig. 4A) reveals two groups. Sequences of populations M1–M3 are in basal position together with one sequence of population AM1 and one sequence of population CM. All remaining sequences are in a derived group with 72 % bootstrap support. The plastid DNA tree (Fig. 4B) also reveals two main groups. One group with 79 % bootstrap support contains sequences of the Afro-Sicilian AFLP group (all but two sequences of populations M1–M3 and all sequences of populations AM1, AM2, and A1–A5). The other group with 81 % bootstrap support contains sequences of the Iberian AFLP group (populations C1–C6, CS and S1–S8) together with one sequence of population M1, one sequence of population M3 and sequences of population CM.

### Hybrids

Differences in the individual trees derived from the different datasets allow identifying individuals of hybrid origin. Individuals of population CM largely correspond to the ‘maroccana’-AFLP group (with two individuals showing admixture from the ‘comosa’-AFLP group), one individual has the more basal ‘maroccana’- and the other the more derived ‘aculeata/comosa/lusitanica’-ribotype, but all individuals have the ‘comosa/lusitanica’-plastid type. Similarly, one individual of population M1 and one individual of population M3 also have the ‘comosa/lusitanica’-plastid type. One individual of population AM1 and both individuals of population AM2 morphologically resemble *H. aculeata* and have the ‘aculeata/comosa/lusitanica’-ribotype, but correspond to the ‘maroccana’-AFLP group. Finally, some individuals of population CS show evidence of admixture by Bayesian admixture clustering of AFLP data. When individuals of putative hybrid origin are excluded, Bayesian inference results in a best-scoring species tree derived from ITS and plastid DNA sequences (Fig. 3B) that is congruent with the species tree derived from AFLP data.

### Ancestral area and age estimation

**Figure 5.**
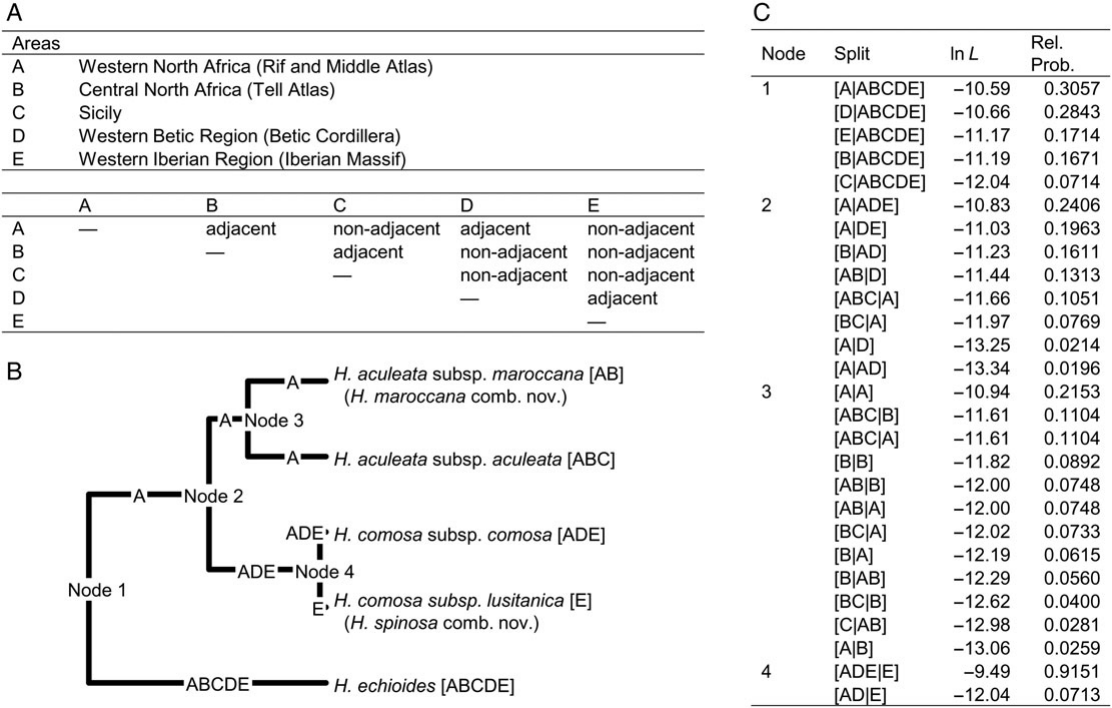
Model-based ancestral area reconstruction in the perennial clade of *Helminthotheca* using the software Lagrange (Ree and Smith 2008). Global −ln *L* at root node is 9.405, the estimated dispersal rate is 1.124, and the estimated local extinction rate is 4.285e−09. (A) Area adjacency matrix used for input. (B) Species tree (consensus tree of *BEAST analysis) displaying most probable ancestral areas. (C) Splits with associated log likelihoods (ln *L*) and relative probabilities (Rel. Prob.) for each node.

Model-based biogeographic reconstruction of ancestral areas (Fig. 5) gives an idea of the biogeographic history of the perennial species of *Helminthotheca*, albeit with little confidence. Western North Africa with its mountain ranges Rif and Middle Atlas is revealed as the ancestral area of the entire clade as well as of the common ancestor of *H. aculeata* subsp. *aculeata* and *H. aculeata* subsp. *maroccana*. The common ancestor of *H. comosa* subsp. *comosa* and *H. comosa* subsp. *lusitanica* is inferred to have lived in an area spanning W North Africa, the W Betic region and the W Iberian region. Molecular clock analysis reveals that the minimum age of the MRCA of the perennial clade dates well to the middle of the Pleistocene (median = 1.09 mya; 95 % HPD interval = 0.48–1.93 mya) **[see** **Supporting Information—Fig. S2****]**.

## Discussion

### Specific entities

Genomic variation as evidenced by Bayesian mixture clustering of AFLP data (Fig. 2B) shows a basic concordance with morphological variation (S. Talavera *et al*., unpubl. data). Diagnostic characters for distinguishing *H. comosa* subsp. *lusitanica* from the nominal subspecies are provided by the involucral bracts of the heads. In the former, the subapical appendix of the inner involucral bracts is shorter (1–3.5 mm long in *H. comosa* subsp. *lusitanica* vs. 5–8 mm long in *H. comosa* subsp. *comosa*) and projects less above the bracts (1–2 mm in *H. comosa* subsp. *lusitanica* vs. 2–6 mm in *H. comosa* subsp. *comosa*). The outer involucral bracts usually have fewer pairs of marginal spines (2–4 in *H. comosa* subsp. *lusitanica* vs. 10–21 in *H. comosa* subsp. *comosa*). Similarly, diagnostic characters for distinguishing *H. aculeata* subsp. *maroccana* from the nominal subspecies are also provided by the involucral bracts of the heads. The outer involucral bracts of *H. aculeata* subsp. *aculeata* usually lack marginal spines. Only rarely do they have one or two pairs of marginal spines in addition to the terminal spine, whereas the outer involucral bracts of *H. aculeata* subsp. *maroccana* have considerably more pairs of marginal spines (10–21).

Following De Queiroz (2007) in treating the existence as separately evolving segments of lineages as the only necessary property of species, we therefore propose to distinguish *H. comosa* subsp. *lusitanica* as a separate species from *H. comosa* and *H. aculeata* subsp. *maroccana* as a separate species from *H. aculeata*. As outlined above, lines of evidence for lineage separation come from AFLP data and morphology. The lineages also have distinct geographic distributions (Fig. 1) and ecological preferences (see below). In this sense, we will refer to *H. comosa* subsp. *lusitanica* as *H. spinosa*, comb. nov., and to *H. aculeata* subsp. *maroccana* as *H. maroccana*, comb. nov., in the following (the new combinations are presented in the section Taxonomic Implications).

### North-western African origin

Although ancestral area reconstruction is not quite conclusive, it suggests that the perennial clade of *Helminthotheca* originated in western North Africa, possibly in the Middle Atlas or Rif. A caveat of the biogeographic analysis, however, is that few populations from central North Africa (Tell Atlas; especially *H. glomerata*) and no populations from the High Atlas and Anti-Atlas have been included, so that we cannot more precisely determine the ancestral area.

Our results for the perennial clade of *Helminthotheca* are well in line with observations in the closely related *Hypochaeris* sect. *Hypochaeris* (Ortiz *et al*. 2009). Both groups originated in western North Africa in the Pleistocene or Pliocene; their origins were thus associated with the ascent of the Mediterranean-type climate and/or Pleistocene climatic oscillations. The importance of the High and Middle Atlas region in addition to the Betic-Rif region for the evolution of Mediterranean plant taxa has been suggested by Médail and Quézel (1997). The High and Middle Atlas Mountains, just as the Rif, are known to have a high rate of endemism, which is explained by the long isolation and high altitude of these massifs, which made them serve as refugium for the Tertiary residual flora. In addition, there is also a large number of schizoendemics that demonstrate the role of the Atlas Mountains in more recent speciation processes (Médail and Quézel 1997). This is in line with climatic reconstructions, which suggest that a Mediterranean-type climate with summer drought was present in northern Africa earlier than in southern Europe (Quézel 1978; Suc 1984). Fauquette *et al*. (1999) describe Pliocene vegetation assemblages resembling the modern thermo-Mediterranean formation for the regions Andalusia (southern Spain), North Africa (Morocco, Algeria, Tunisia) and Sicily, corresponding to those regions inhabited by perennial species of *Helminthotheca*. In accordance with our age estimates, we might hypothesize that after the common ancestor of the perennial clade of *Helminthotheca* had originated in response to the ascent of the Mediterranean-type climate in northern Africa, it held on during the Pliocene and diversified in the Pleistocene.

### Migratory routes out of western North Africa

The evolutionary scenario derived from our data suggests two migratory routes out of western North Africa and across the Mediterranean Sea. The first expansion was to the north, across the Strait of Gibraltar, to the Iberian Peninsula. The second expansion was to the east, along central North African territories, to Sicily and the Apennine Peninsula.

Numerous studies have investigated genetic differentiation between populations on both sides of the Strait of Gibraltar (e.g. Arroyo *et al*. 2008; Escudero *et al*. 2008), with differing outcomes. Whereas the Strait of Gibraltar has been an effective barrier to gene flow in *Carex helodes* after its dispersal from the Iberian Peninsula to North Africa (Escudero *et al*. 2008), extensive gene flow across the Strait of Gibraltar via seed dispersal has been documented in *Calicotome villosa* (Arroyo *et al*. 2008). In the central Mediterranean region, the Strait of Sicily separating North Africa and Sicily has also functioned as an important route of dispersal for plants and animals (e.g. Habel *et al*. 2008; Fernández-Mazuecos and Vargas 2011). Dispersal was probably facilitated by lowered sea water levels during the Pleistocene glacial periods (Thiede 1978). In *Helminthotheca*, seed dispersal could have been effectuated by wind or migratory birds (Finlayson 1992).

### Ecogeographic isolation

As in other Mediterranean plant groups of Pliocene/Pleistocene age such as *Antirrhinum* (Vargas *et al*. 2009) and *Reseda* sect. *Glaucoreseda* (Martín-Bravo *et al*. 2010), there is also a clear geographic component to speciation in the perennial clade of *Helminthotheca*. The biogeographic analysis suggests two lineage divergence events within western North Africa, the first yielding an Afro-Sicilian and an Afro-Iberian lineage and the second yielding *H. maroccana* (as progenitor) and *H. aculeata* (as derivative) within the Afro-Sicilian lineage. The placement of *H. glomerata* in this scenario still has to be determined. The third lineage divergence event is situated in the western Iberian region, yielding *H. comosa* (as progenitor) and *H. spinosa* (as derivative).

Accurate identification of species pairs is crucial for investigating isolating barriers. The limit between the distributions of *H. comosa* and *H. spinosa* closely follows the Guadalquivir river basin, which separates the Mesozoic and Cenozoic Betic Cordillera (‘Western Betic Region’; García-Barros *et al*. 2002) inhabited by *H. comosa* and the Paleozoic Iberian Massif (‘Western Iberia’) inhabited by *H. spinosa*. Characteristics of the two regions such as those associated with the substrate (calcareous in the Betic Cordillera, acidic in the Iberian Massif) are expected to exert divergent selective pressure on populations, resulting in ecogeographic isolation of the diverging lineages (Sobel *et al*. 2010). Ecogeographic isolation might also be hypothesized for the species pair *H. aculeata*/*H. maroccana*, in response to differences in substrate or precipitation. *Helminthotheca aculeata* grows on calcareous substrate of the Cenozoic, whereas *H. maroccana* grows on crystalline rocks and schist. Hybrids exist in contact zones between the regions. For example, some populations of *H. comosa* grow in calcareous enclaves, which exist in the western Iberian region. Hybrids between *H. spinosa* and *H. comosa* are found precisely in the ecotones between acidic and calcareous soils.

As already supposed by Sauvage (1961), assessment of ploidy levels could shed more light on the issue of hybridization coupled with polyploidization in *Helminthotheca*. To the present day, there are few chromosome counts available, whereby a diploid chromosome number (2*n* = 10) has been reported for *H. comosa* and *H. spinosa* (Fernandes and Queirós 1971; Gallego 1981; Luque 1981). A *H. comosa*-like tetraploid population (2*n* = 20) has been found in northern Morocco (*Talavera et al. 327/03*, SEV237787; S. Talavera, unpubl. data), close to the here analysed hybrid population CM.

### Taxonomic implications

As a result of the genetic analyses and the morphological survey of the populations included in this work (S. Talavera *et al*., unpubl. data), two new combinations and descriptions of three hybrids that are new to science are now necessary.

#### New combinations

Helminthotheca maroccana *(Sauvage) Talavera & Tremetsberger, comb. nov.: Picris aculeata* subsp. *maroccana* Sauvage in Trav. Inst. Sci. Chérifien, Sér. Bot. 22: 202 (1961) ≡ *Helminthotheca aculeata* subsp. *maroccana* (Sauvage) Greuter in Willdenowia 33: 233 (2003). Ind. loc.: ‘Forêt des Bni-Âbid.’ Holotype: herbarium C.S. [Charles Sauvage] no. 14689.

Helminthotheca spinosa *(DC.) Talavera & Tremetsberger, comb. nov.: Helminthia spinosa* DC. in Candolle & Lamarck, Fl. Franç., ed. 3, 4: 58 (1805) ≡ *Picris spinosa* (DC.) Poir. in Lamarck, Encyc., Suppl. 4: 408 (1816). Ind. loc.: ‘Je décrit cette plante d'après des échantillons originaires des Pyrénées et qui proviennent de l'herbier de Lemonnier’. Lectotype, here designated: G00317181 [G-DC]. The type material that is conserved consists of the upper part of three plants, mounted on two ‘échantillons’ or vouchers. One of the vouchers comprises two plants and the other one plant. In the voucher with two plants, there are also two handwritten labels. On one label, which possibly stems from the herbarium of Lemonnier, it is indicated ‘Picris aculeata Desf.?/P. sprengeriana Gaert.?/Pyrénées’, and on the other label, A. P. de Candolle indicated ‘Helminthia spinosa Fl. Fr.’. The other voucher, with a single plant, does not contain any label, but the plant is similar to the other two. We select as lectotype the plant mounted on the right side of the voucher that contains the two labels, the more complete of the two plants. The other plant of this voucher and the plant from the other voucher are isolectotypes.

=*Helminthotheca comosa* subsp. *lusitanica* (Welw. ex Schltdl.) P. Silva & Escud. in Bol. Soc. Brot., sér. 2, 60: 156 (1987) [for synonyms see Greuter, Med-Checkl. 2: 240 (2008)].

#### New hybrids

Helminthotheca × hispanica *Tremetsberger & Talavera*, hybr. nov.: Helminthotheca comosa (Boiss.) Holub × *H. spinosa* (DC.) Talavera & Tremetsberger

Holotype: SEV237782. Spain, Huelva, Hinojos, ‘Las Porqueras’, in *Quercus suber* forest, 80 m, 37.29°N-6.42°W, 30 June 2007, S. Talavera, n° 228/07. Isotypes: SEV237783, SEV237784.

Plants with leaves that surround the involucre (outer involucral bracts) similar to those of *H. comosa*, but the subapical appendix of the inner involucral bracts is similar in length to that of *H. spinosa* (5–8 mm long in *H. comosa*; 1–3.5 mm long in *H. spinosa*; 2–3 mm long in *H.* × *hispanica*).

Helminthotheca × tingitana *Tremetsberger & Talavera, hybr. nov.:* Helminthotheca comosa (Boiss.) Holub × *H. maroccana* (Sauvage) Talavera & Tremetsberger

Holotype: SEV256853. Morocco, Tangier-Tétouan, Chefchaouen, pr. Aguelman, carretera de Chefchaouen a Tetuán, 536 m, 35°22′08″N-5°22′38.2″W, 17 June 2008, E. Rico et al., n° MS-999. Isotypes: MA779452, SALA132080.

Plants with the subapical appendix of the inner involucral bracts (6.5–7 mm long) similar to that of *H. comosa*, but the leaves that surround the involucre (outer involucral bracts) have more than 50 spines in the margin, similar to those of *H. maroccana*.

Helminthotheca × riphaea *Tremetsberger & Talavera, hybr. nov.:* Helminthotheca aculeata (Vahl) Lack × *H. maroccana* (Sauvage) Talavera & Tremetsberger

Holotype: SEV256845. Morocco, Taza-Al Hoceima-Taounate, pr. Ait Isa, en la carretera de Targuist a Al Hoceima, 1012 m, 35°1′27.2″N-4°10′4″W, 18 June 2008, E. Rico et al., n° MS-1099. Isotypes: MA782913, SALA132081.

Plants morphologically more similar to *H. aculeata* than to *H. maroccana*, but the leaves that surround the involucre (outer involucral bracts) have 2–7 pairs of marginal spines and some of the peduncles are doliform in the apex, as in *H. aculeata*, and some are cylindric, as in *H. maroccana*.

## Conclusions

This study demonstrates that phylogeography above the species level is a powerful tool for investigating patterns and processes at the boundary between divergent and reticulate relationships. The western Mediterranean region, and specifically western North Africa, is highlighted as a region of intensive recent speciation. The inferred evolutionary history is compatible with the concept of ecogeographic isolation, which refers to the fact that geographic ranges of diverging lineages are largely non-overlapping due to adaptive differentiation (Sobel *et al*. 2010).

## Accession Numbers

All sequences obtained in this study have been deposited in the EMBL Nucleotide Sequence Database under the accession numbers LN830755–LN830895.

## Sources of Funding

This work was supported by a Juan de la Cierva fellowship [to K.T.] and projects of the Ministerio de Educación y Ciencia resp. Ministerio de Ciencia e Innovación resp. Ministerio de Economía y Competitividad [Spain; grant numbers CGL2009-08178, CGL2012-32914, CGL2008-02486-E and CGL2008-02531-E].

## Contributions by the Authors

S.T. conceived the idea of the paper, F.B., R.C.-S., M.Á.O, M.T., S.T., A.T., and K.T. collected plant material, M.T. and S.T. studied herbarium material, A.T. and K.T. performed the analyses and K.T. led the writing of the manuscript.

## Conflict of Interest Statement

None declared.

## Supporting Information

The following additional information is available in the online version of this article —

**Figure S1.** Common secondary structure of ITS2 rRNA of *Helminthotheca*.

**Figure S2.** Chronogram of *Helminthotheca* and the two closest outgroup genera, *Leontodon* and *Picris*, based on ITS1 and ITS2 sequences.

Additional Information
