# Supplementary material for: Phylogeography above the species level for perennial species in a composite genus
Source: AoB Plants. 2015 Dec 7;8:plv142. doi: 10.1093/aobpla/plv142 (PMC4720837; doi:10.1093/aobpla/plv142)
Supplement: Additional Information [file supp_8_plv142_index.html]

Phylogeography above the species level for perennial species in a composite genus — Phylogeography above the species level for perennial species in a composite genus — Additional Information 

# Phylogeography above the species level for perennial species in a composite genus

## Additional Information

Additional Information

- Additional Information - Docx file
- Supplementary file1 - jpg file
- Supplementary file2 - jpg file
- Supplementary file4 - jpg file
