# Supplementary material for: Phylogeography above the species level for perennial species in a composite genus
Source: AoB Plants. 2015 Dec 7;8:plv142. doi: 10.1093/aobpla/plv142 (PMC4720837; doi:10.1093/aobpla/plv142)
Supplement: Additional Information [file supp_plv142_plv142supp.docx]

**SUPPORTING INFORMATION**

The following [**SUPPORTING INFORMATION**] is available in the online version of this article:

**File 1.** Figure. Common secondary structure of ITS2 rRNA of *Helminthotheca*.

**File 2.** Figure. Chronogram of *Helminthotheca* and the two closest outgroup genera, *Leontodon* and *Picris*, based on ITS1 and ITS2 sequences.
