# Supplementary figures and images for: Phylogeography above the species level for perennial species in a composite genus
Source: AoB Plants. 2015 Dec 7;8:plv142. doi: 10.1093/aobpla/plv142 (PMC4720837; doi:10.1093/aobpla/plv142)

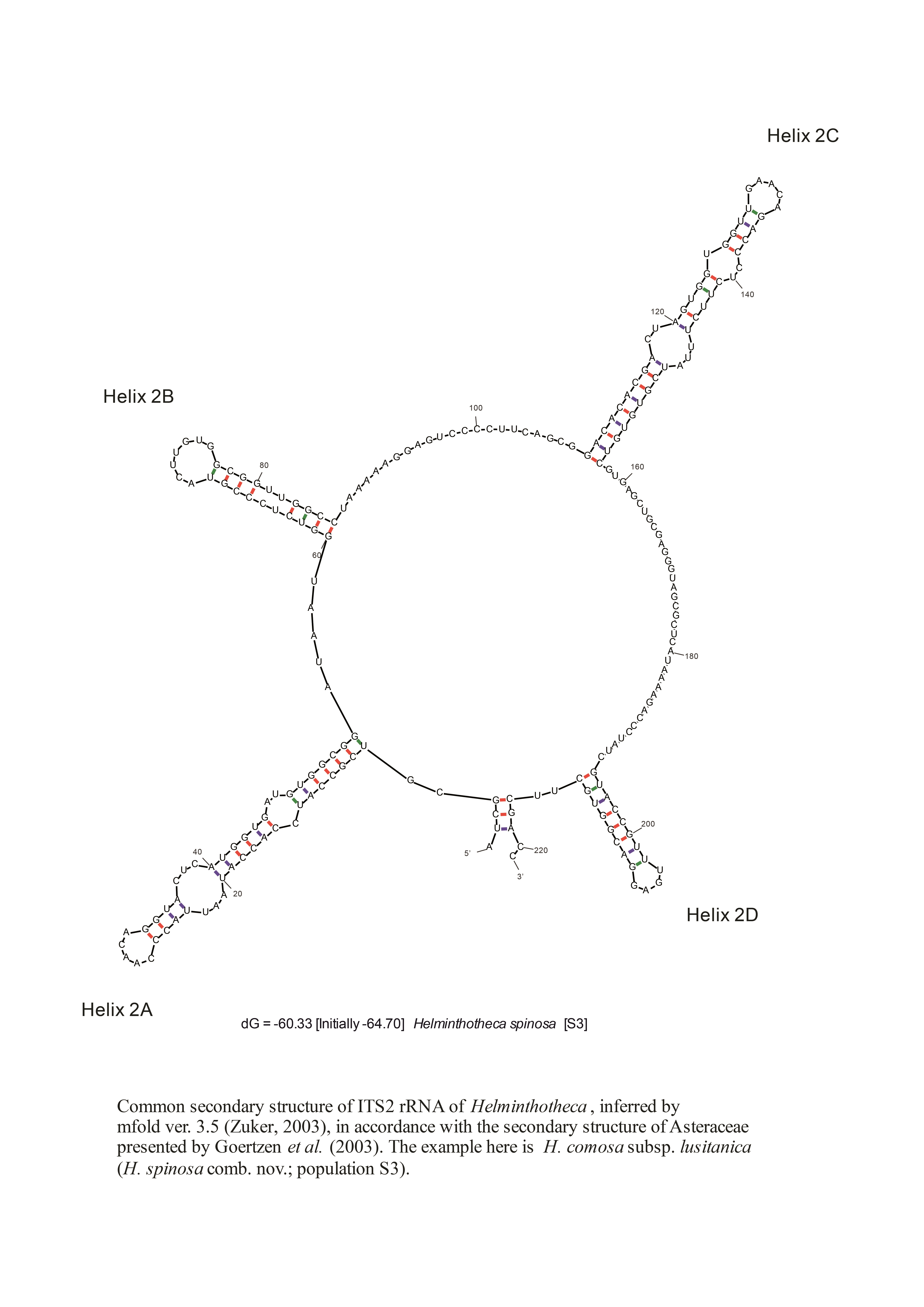

Supplement: Additional Information [file supp_plv142_plv142supp_file1.jpg]

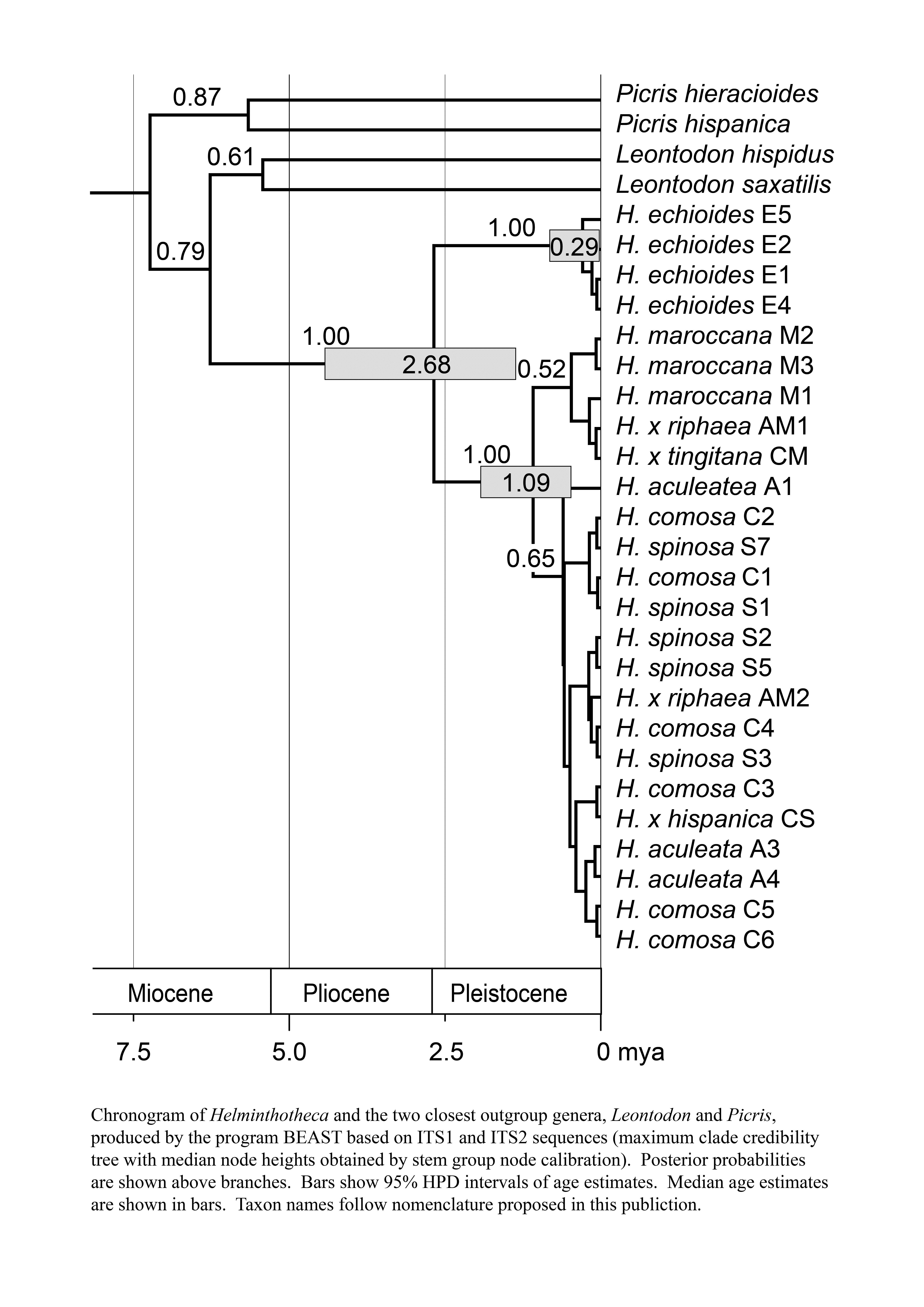

Supplement: Additional Information [file supp_plv142_plv142supp_file2.jpg]

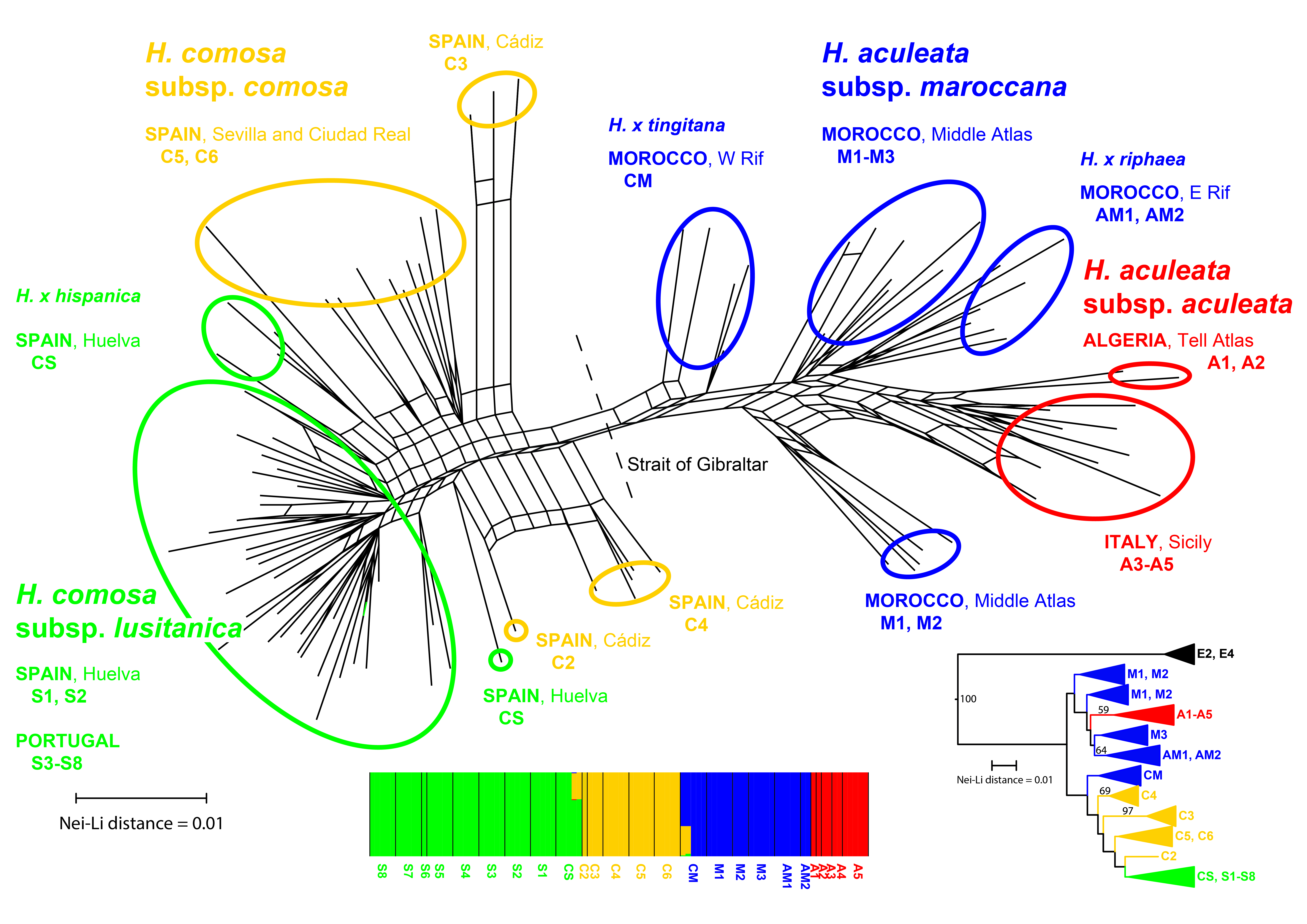

Supplement: Additional Information [file supp_plv142_plv142supp_file4.jpg]
